# What is the association between childhood adversity and subsequent chronic pain in adulthood? A systematic review

**DOI:** 10.1016/j.bjao.2023.100139

**Published:** 2023-06-07

**Authors:** Karen P. Nicolson, Sarah E.E. Mills, Dhaneesha N.S. Senaratne, Lesley A. Colvin, Blair H. Smith

**Affiliations:** 1 Division of Population Health & Genomics, University of Dundee, Dundee, UK; 2 School of Medicine, University of St Andrews, St Andrews, UK

**Keywords:** adverse childhood experiences, childhood adversity, childhood trauma, chronic pain, systematic review

## Abstract

**Background:**

Adverse childhood experiences and chronic pain are complex problems affecting millions of people worldwide, and result in significant healthcare utilisation. Our review aimed to determine known associations between adversity in childhood and chronic pain in adulthood.

**Methods:**

We performed a prospectively registered systematic review (PROSPERO ID: 135625). Six electronic databases (Pubmed, Medline, Cochrane, Scopus, APA PsycNet, Web of Science) were searched from January 1, 2009 until May 30, 2022. Titles and abstracts were screened, and all original research studies examining associations between adverse childhood experiences and chronic pain in adulthood were considered for inclusion. Full texts were reviewed, and a narrative synthesis was used to identify themes from extracted data. Ten percent of studies were dual reviewed to assess inter-rater reliability. Quality assessment of study methodology was undertaken using recognised tools.

**Results:**

Sixty-eight eligible studies describing 196 130 participants were included. Studies covered 15 different types of childhood adversity and 10 different chronic pain diagnoses. Dual reviewed papers had a Cohen's kappa reliability rating of 0.71. Most studies were of retrospective nature and of good quality. There were consistent associations between adverse childhood experiences and chronic pain in adulthood, with a ‘dose’-dependent relationship. Poor mental health was found to mediate the detrimental connection between adverse childhood experiences and chronic pain.

**Conclusion:**

A strong association was found between adverse childhood experiences and chronic pain in adulthood. Adverse childhood experiences should be considered in patient assessment, and early intervention to prevent adverse childhood experiences may help reduce the genesis of chronic pain. Further research into assessment and interventions to address adverse childhood experiences is needed.

Chronic pain and adverse childhood experiences are common and complex issues globally. Adverse events in childhood have been shown to be detrimental to adult mental and physical well-being,^1^^,^^2^ with lifelong consequences.^3^ Adverse childhood experiences may be defined as traumatic experiences occurring before the age of 18, and include (but are not limited to) physical/emotional/sexual abuse, physical/emotional neglect, and familial/household dysfunction (e.g. parental incarceration, parental mental health disorder, parental separation).^4^ Adverse childhood experiences have been called a public health crisis, and it is widely accepted that they have a detrimental impact on social and health outcomes.^5^^,^^6^ In the UK, 47% of people were found to have lived through, or with, at least one type of adverse childhood experience; however, 12.3% had lived with four or more.^7^ WHO data from a Mental Health Survey of nine countries showed that the mean number of adverse childhood experiences is 2.5–2.9.^4^^,^^8^^,^^9^

Chronic pain is a major long-term condition, affecting 19% of adults in Europe.^10^ In the UK, chronic pain affects between a third and a half of all adults, approximately 28 million people.^11^ Chronic pain is pain that persists or recurs for longer than 3 months.^12^^,^^13^ Rather than being considered a symptom of other diseases, chronic pain is a distinct clinical entity and is a diagnosis in its own right, recognised in the most recent International Classification of Diseases, 11th Revision (ICD-11) classification.^14^ It is associated with poor general health and quality of life, and has detrimental impacts on financial stability, relationship dynamics, and healthcare utilisation; similarly, people who have experienced adverse childhood experiences have also been shown to have high levels of healthcare utilisation.^15^^,^^16^

Previous systematic reviews have analysed chronic pain experience and previously self-reported childhood abuse, concluding that there was an increased risk of chronic pain in those who had experienced abuse and neglect.^17^ Other reviews have focused on a specific type of chronic pain^18^^,^^19^ or a specific type of adverse childhood experience.^20^ This current systematic review and narrative synthesis aimed to identify and describe associations between any adverse childhood experience and any/all types of chronic pain reported in adulthood (i.e. after the age of 18).

## Methods

Childhood adversity is described and classified in many ways throughout the literature. For the purposes of this review, we defined ‘adverse childhood experiences’ as traumatic experiences endured by children before the age of 18 yr.^4^^,^^21^^,^^22^ The term ‘chronic pain’ was used to capture any type of chronic pain that had been studied alongside any adverse childhood experience. Although recognising that chronic pain is a heterogeneous condition, for the purposes of this paper all forms of chronic pain were considered together. The review was prospectively registered with PROSPERO (ID: 135625).

### Search strategy

To optimise study identification, search strategies were developed by the research team with librarian support. Electronic databases (Pubmed, Medline, Cochrane, Scopus, APA PsycNet, Web of Science) were searched for papers published from 1 January 2009 until 30 May 2022. Further studies were identified by hand searching bibliographies of any relevant systematic reviews and searching tables of contents of relevant journals for articles published in the same time period. The search terms used were:

1. Child OR childhood and child/childhood abuse and/or childhood maltreatment and/or child/childhood adversity and/or childhood trauma and/or childhood neglect;
2. Adult survivors of abuse;
3. ‘Chronic Pain’; ‘Long-term pain’; ‘Physical suffering’; ‘Persistent Pain’
4. 1 AND 2 AND 3.

### Inclusion/exclusion criteria

Studies were eligible for inclusion if they included at least one type of adverse childhood experience and examined possible biological, psychological, or social associations between these adversities and the subsequent presence of chronic pain in adulthood (aged 18 yr or older). No limits were applied for country of origin, type of adverse childhood experience, type of chronic pain, or study design. Articles published in languages other than English were excluded owing to lack of access to translation services. Articles for which no full-text version was available were also excluded. Review articles and any papers reporting duplicate data/studies were excluded, although the referenced original source papers were reviewed for inclusion.

### Screening and data extraction

From the initial longlist, study titles and abstracts were independently screened by two reviewers (KPN and SEEM). Full text review was performed by KPN, and 10% of included studies were double-reviewed by DS to assess inter-rater reliability. Disagreements were resolved through discussion with BHS.

The following data were extracted using a standardised form: country of origin, cohort size, demography, type of chronic pain, type of childhood adversity, questionnaire type used to determine the adversity experienced, primary and secondary adverse outcomes reported, association with adversity, conclusions, and key concepts.

### Narrative synthesis

A narrative synthesis was used to identify common themes reflecting the associations. Two reviewers (KPN and SEEM/DS) independently identified thematic associations between childhood adversity and chronic pain. Emerging themes were combined iteratively into a narrative synthesis to summarise and describe the findings of included studies. Decisions on identification and grouping of themes were agreed by all authors.

### Quality assessment

Each study's risk of bias was independently assessed by two reviewers (KPN and SEEM/DS) using standardised tools selected for each study design. Tools used for quality assessment of included papers were selected from the tools recommended by the Scottish Intercollegiate Guideline Network (SIGN).^23^ SIGN is an internationally recognised organisation that produces evidence-based clinical guidelines and is accredited as part of the Guidelines International Network. AXIS was chosen as the best available tool for cross-sectional studies, as SIGN does not provide a tool for this study design. The quality assessment tools used in this analysis were: cross-sectional studies – AXIS appraisal tool^24^; cohort studies – SIGN methodology checklist tool 3^23^; case-control studies – SIGN methodology checklist tool 4.^23^

## Results

### Overview of results

**Fig 1:**
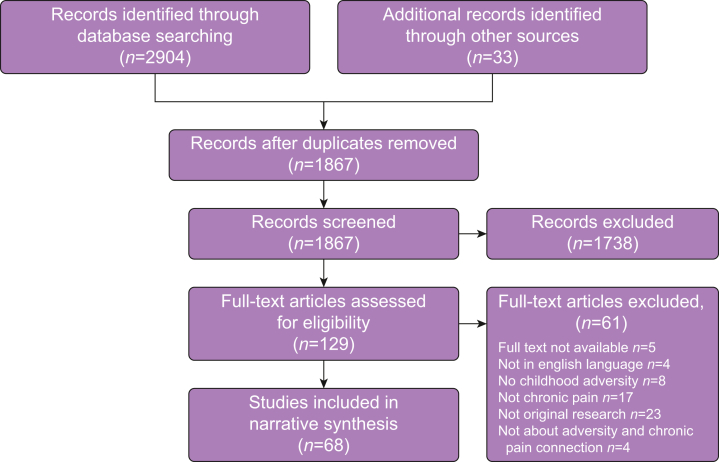
PRISMA diagram of systematic search results. PRISMA, Preferred Reporting Items for Systematic Reviews and Meta-Analyses.

We identified 2904 records from the search strategy, and 33 records were identified through other sources (Fig 1). Titles, abstracts, and full-text papers were screened, identifying 68 studies for inclusion with data from 196 130 participants. There were 50 cross-sectional studies, 14 case-control studies, and four cohort studies: 10% of studies were double-reviewed for inter-rater reliability with Cohen's kappa of 0.71. Demographic information from all included studies is shown in Supplementary Table S1.

**Fig 2:**
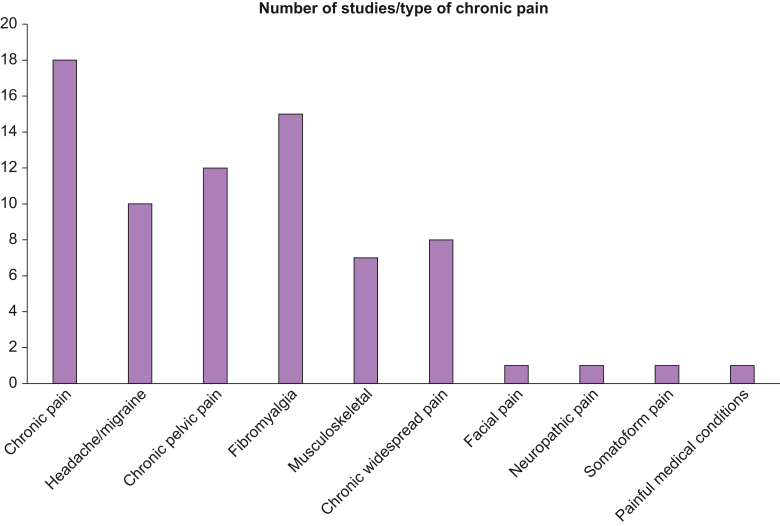
Number of studies describing each type of chronic pain, for studies included in the systematic review and narrative synthesis.

The combination of emotional/physical/sexual abuse was the most commonly studied adverse childhood experience, followed by general ‘adverse childhood experiences’, and ‘physical and sexual abuse’ as shown in Table 1. Of the 10 types of chronic pain identified in included studies as being associated with adverse childhood experiences, generalised chronic pain, fibromyalgia, and chronic pelvic pain were the most common (Fig 2). Supplementary Table S2 demonstrates the impact of childhood adversity findings from each study and the recommended outcomes. The main themes identified in this narrative synthesis, with the corresponding evidence sources, are summarised in Table 2.

**Table 1:** Type of childhood adversity documented in study.

Type of childhood adversity	Number of studies	Authors and years of studies
Emotional/physical/sexual abuse	33	Gerber and colleagues (2018),^25^ Gunduz and colleagues (2018),^26^ Karas and colleagues (2017),^27^ Riedl and colleagues (2019),^28^ Schrepf and colleagues (2018),^29^ Poli-Neto and colleagues (2018),^30^ Coppens and colleagues (2017),^31^ Eriksen and colleagues (2016),^32^ Brown and colleagues (2018),^33^ Generaal and colleagues (2016),^34^ Tietjen and colleagues (2010),^35^ Bohn and colleagues (2013),^36^ Fishbain and colleagues (2014),^37^ Hauser and colleagues (2019),^38^ De Roa and colleagues (2018),^39^ Prangnell and colleagues (2020),^40^ Kascakova and colleagues (2020),^41^ Bayram and Erol (2014),^42^ Tietjen and colleagues (2010),^43^ Macedo and colleagues (2019),^44^ Alhalal and colleagues (2018),^45^ Coppens and colleagues (2018),^46^ Achenbach and colleagues (2022),^47^ Yeung and colleagues (2016),^48^ Häuser and colleagues (2012),^49^ Liebermann and colleagues (2018),^50^ Maatta and colleagues (2019),^51^ Nicolson and colleagues (2010),^52^ Powers and colleagues (2014),^53^ Tesarz and colleagues (2016),^54^ Tietjen and colleagues (2010)^55^
Parental attachment/affection/bonding	1	Anno and colleagues (2015)^56^
Physical abuse	1	As-Sanie and colleagues (2014)^57^
Traumatic experience, physical abuse, and emotional abuse	1	Bottiroli and colleagues (2018)^58^
Emotional, physical, and sexual abuse + Violence (Witnessing and Domestic)	2	Chiu and colleagues (2017),^59^ Santo and colleagues (2022)^60^
Adverse childhood experiences	3	Sachs-Ericsson and colleagues (2017),^61^ Anda and colleagues (2010),^62^ Fowler and colleagues (2020)^63^
Childhood adversities	4	Scott and colleagues (2011),^64^ Salonsalmi and colleagues (2021),^65^ Varinen and colleagues (2017),^66^ Piontek and colleagues (2021)^67^
Physical and sexual abuse, parental mental health, poverty, and family function	1	Gonzalez and colleagues (2012)^68^
Hospitalisation, surgical operations, maternal separation, parental death, family difficulties	1	Jones and colleagues (2009)^69^
Sexual abuse	1	Kamiya and colleagues (2016)^70^
Sexual abuse, punishment, and neglect	1	Johnson and colleagues (2020)^71^
Physical + sexual abuse and fear of danger	1	Khandker and colleagues (2014)^72^
Physical and sexual abuse	4	Harris and colleagues (2018),^73^ Taghian and colleagues (2021),^74^ McBeth and colleagues (2015),^75^ Hart-Johnson and colleagues (2012)^76^
Serious illness before age 24 yr	1	Muthuri and colleagues (2016)^77^
Emotional/physical/sexual abuse and primary care giver attachment	2	Nacak and colleagues (2017),^78^ Waller and colleagues (2016)^79^
Emotional/physical/sexual abuse and household dysfunction	1	McKernan and colleagues (2019)^80^
Traumatic events	1	Naliboff and colleagues (2015)^81^
Emotional/physical/sexual abuse and bullying	1	Nault and colleagues (2016)^82^
Emotional/physical abuse	1	Ortiz and colleagues (2016)^83^
Sexual/emotional/physical abuse and family/household dysfunction, and parental ill health	2	Stickley and colleagues (2015),^84^ Reuchlein and colleagues (2016)^85^
Emotional abuse	1	Saariaho and colleagues (2011)^86^
Emotional/physical/sexual abuse and traumatic events	2	Schrepf and colleagues (2018),^29^ You and colleagues (2019)^87^
Traumatic events, road traffic accidents, natural disasters, witnessing death, child abuse	1	Smith and colleagues (2010)^88^
Bullying in childhood	1	Varinen and colleagues (2019)^89^
Perceived childhood neglect	1	Ziadni and colleagues (2020)^90^

**Table 2:** Summary of main themes.

Main findings from study	Study reference	Number of studies
Greater number of adversities are associated with worse pain experience.	25, 26, 27, 29, 30, 31, 32, 33, 34, 35, 56, 58, 60, 61, 63, 64, 69, [Bibr bib87][Bibr bib45][Bibr bib49][Bibr bib50][Bibr bib52][Bibr bib57], 57, 58,42, 75, 41,45, 45, 45, 45, 45, 45, 45, 45, 45	46
Current poor mental health mediates association between childhood adversity and pain.	^30^,^31^,^72^,^42^,^45^,^93^,^50^,^53^	8
In those with chronic pain, previous childhood adversity should be assessed.	^26,^^28^, ^29^, [Bibr bib57][Bibr bib61][Bibr bib71][Bibr bib39][Bibr bib74][Bibr bib60][Bibr bib61][Bibr bib62][Bibr bib67][Bibr bib45][Bibr bib84][Bibr bib91][Bibr bib82][Bibr bib50], 76, 93,^52^,^85^,^92^	23
Screening for mental health should be considered for those with chronic pain.	^57^,^31^,^39^,^41^,^42^,^67^,^45^,^80^,^82^,^93^,^70^	9
Education on the awareness of childhood adversity on pain needs to be more widespread.	^42^^,^^53^	2
Early intervention is key to prevent childhood adversities from occurring.	^28^^,^^65^^,^^66^^,^^89^^,^^94^^,^^68^	6

### Quality assessment

Sixty-one of the 68 included studies were assessed as high quality. Seven papers were assessed as having moderate risk of bias. Elements of weakness that were identified in quality analysis included small sample size (11 studies had fewer than 100 participants); limited demographic information; exact age of participants was missing (13 studies); and further details on breakdown of gender was not provided (seven studies).

Most papers were consistent in noting recall bias as a potential weakness. The main confounder documented was mental health in the form of anxiety, depression, current stress, and post-traumatic stress disorder (PTSD). In cross-sectional studies, a common weakness was lack of information about non-responders. One paper lacked analysis to determine the statistical significance of associations reported; however, it did not add any factors that had not been identified by other studies included in the review.^25^ Further details of quality assessment are available in Appendix 1.

### Narrative synthesis

Adverse childhood experiences were shown to increase the risk of subsequent chronic pain as an adult and in one study, 75% of participants with chronic pain had experienced early life adversity.^26^ Childhood violence was associated with multiple physical diseases, of which chronic pain was the most prevalent.^72^, ^27^, ^28^ Adverse childhood experiences were shown to lead to greater dysfunction and disability for those with chronic pain when compared with healthy controls, or those with pain but no adversity.^29^^,^^57^ The more adversity experienced in childhood, the more likely an adult was to experience chronic pain, and the greater the chance that the chronic pain would not improve^29^^,^^30^, ^31^, ^63^, ^87^ adding to the chronicity of pain.^59^ Specific adverse childhood experiences in the form of parental loss early in life, along with verbal and sexual abuse were independently associated with the subsequent onset of painful conditions. Adverse childhood experiences led to a higher risk of developing mental health and painful conditions.^64^^,^^61^ Those who experienced adverse childhood experiences were shown to have more sites of chronic pain and pain of greater severity than those who did not experience such adversity.^32^, ^33^, ^34^, ^35^, ^61^, ^64^ However, although stress that is experienced in childhood is associated with increased risk of developing chronic pain in adulthood, stress that is experienced in adulthood does not show the same correlation with developing chronic pain.^71^ Those with chronic pain who experienced adverse childhood experiences had greater sensitivity and a lower tolerance to pain than those without a history of adverse childhood experiences.^87^ ‘Catastrophising’ and hypervigilance to somatic symptoms in chronic pain were also found to be related to previous adverse childhood experiences.^36^^,^^95^ Two studies did not find a connection between adverse childhood experiences and chronic pain; however, each of these studies concentrated on only one form of adversity and type of pain.^37^^,^^38^

An insecure environment where children were emotionally deprived was also related to chronic pain development. Those who experienced emotional neglect, affectionless parenting, parental loss, or overprotective parenting in childhood were more likely to experience chronic pain.^30^^,^^39^, ^55^, ^86^ A combination of adverse childhood experiences, ‘alexithymia’ (an inability to determine the cause of feelings in your body) and ongoing life stress may lead to chronic pain.^58^^,^^78^ Somatoform pain disorder was also linked to previous emotional neglect, poor awareness of body signals in the context of depression, and poor attachment to primary caregiver.^87^ Emotional abuse was related to higher pain sensitisation and was found to be associated with chronic pain in people who injected drugs.^40^ In the population of people who inject drugs, physical and sexual abuse led to doubling of risk of developing chronic pain in adulthood. In those with substance misuse, pain severity was greater. ‘Catastrophisation’ and detrimental pain interference in life was also more prevalent for those who had experienced physical and sexual abuse when with those without these particular adversities.^74^ In those with chronic pain, a larger number of adverse childhood experiences were found to lead to a greater risk of substance misuse and mental health disorders compared with those with fewer adverse childhood experiences.^60^ Living in a house with financial strain; experiencing parental divorce, parental mental ill health, or alcohol problems; and/or living in fear of a family member were associated with a greater chance of developing chronic pain in adulthood compared with those with no adversity.^66^ Poor social situations, physically traumatic events such as accidents, and living in fear during childhood were also associated with chronic pain.^69^^,^^72^ Experiencing bullying as a child may be related to chronic pain, either directly or mediated by depression.^33^^,^^87^

Current poor mental health as an adult is thought to mediate the development and severity of chronic pain in the presence of childhood adversity,^41^ particularly with depression and anxiety.^39^^,^^41^, ^41^, ^66^ Borderline personality disorder and childhood adversity were also associated with chronic pain; childhood adversity was associated with experiencing pain and particular pain syndromes such as fibromyalgia in those with borderline personality disorder.^71^ However, even after controlling for mental health, the dose–response relationship between adverse childhood experiences and subsequent chronic pain persisted.^61^ Depression can worsen an experience of pain,^62^ and those with depression and pain were found to have experienced more emotional neglect than those who had depression and no chronic pain.^27^^,^^44^ Anxiety may be caused by adverse childhood experiences which in turn contributes to chronic pain prevalence.^94^ PTSD was found to play a part in chronic pain experience^45^^,^^80^ causing a lower level of functioning and higher levels of pain when adverse childhood experiences had been experienced. Perceived stress and injustice as an adult have been found to mediate the relationship between chronic pain and adverse childhood experiences.^88^^,^^90^

A few studies explored potential biological mechanisms driving the identified associations. The direct physiological changes caused by adverse childhood experiences that influence chronic pain are still debated. Hypotheses include disruption in cortisol regulation or that maternal neglect causes neurobiological changes.^31^^,^^42^ Irregularities of the hypothalamic pituitary axis have also been postulated, whereas leptin and endocannabinoid dysfunction after adversity may be related.^46^^,^^47^ An association was also found connecting serious illness, social adversity, and physical trauma before the age of 25 to chronic widespread pain, more than chronic regional pain, raising the possibility that different mechanisms may underlie different types of chronic pain. Central sensitisation leading to pain sensitivity and longevity has also been postulated.^87^

## Discussion

### Summary

Studies in this review consistently documented high levels of adverse childhood experiences in adults with chronic pain. Many studies used a pre-existing formal chronic pain diagnosis as an inclusion criterion, rather than asking specifically about any pain lasting longer than 3 months*.* A single type of adversity was rarely found in isolation; where there was one form of adversity, there were usually others.^57^^,^^56^^,^^84^ This review shows that adverse childhood experiences, in its many forms, can have a detrimental impact in the form, presence, severity, and extent of chronic pain, the greater number of adversities experience the worse this effect is.

### Strengths and limitations

This is the first comprehensive systematic review to describe known associations between any form of adverse childhood experience and any type of adult chronic pain. Strengths include detailed analysis of a wide range of studies from many different countries covering a wide range of adverse childhood experiences and types of chronic pain. Limitations include exclusion of papers not available in full or in English (for reasons of resource), which may produce a cultural and geographical bias; however, only four papers were excluded for this reason, so the impact of this limitation is likely to have been minimal. Further limitations include terminology for adverse childhood experiences; the heterogeneity of adversity a child can experience and consequent range of descriptive terms, means studies may have been missed. We used a broad search strategy across multiple databases, and extensive hand-searching to supplement the initial search strategy, so we hope to have minimised the risk of missing relevant studies. However, given the heterogeneity of terminology in this field, we cannot be certain that a small number of relevant studies were not missed. The majority of studies were retrospective; therefore, recall bias was also noted. This is a limitation of the methods used to address the research questions. Most of the studies included were of high quality, with only six noted to be of medium quality and two of poor quality. Any studies of poorer methodological quality were included in the final synthesis for completeness, and although their conclusions were given less emphasis in the final synthesis, their findings were consistent with higher quality studies (see Table 2). The complexity and heterogeneity of data meant that we opted for a narrative analysis. If consistent definitions and measurements of adverse childhood experiences can be developed, then future work could involve substantial meta-analysis.

### Comparison with existing literature

The more types of adverse childhood experiences, the greater the detrimental impact on development and experience of pain in adult life. However, much of the published research has a relatively narrow focus on emotional, physical, and sexual abuse. In previous systematic reviews, fibromyalgia has been shown to be associated with prior psychological or physical trauma.^19^ A meta-analysis examining only two types of adversity (physical or sexual abuse) found that those who recalled adverse childhood experiences were at greater risk of chronic pain as an adult when compared with those who had not reported experiencing such events in their childhood.^17^ In addition, a previous meta-analysis found that sexual abuse was linked to non-specific chronic pain and chronic pelvic pain.^96^ Poor mental health may be a contributory factor to chronic pain, and the negative impact of childhood adversity on mental health is already documented.^97^^,^^96^ This current systematic review examines associations with a much broader range of adversities such as household dysfunction, experiencing bullying, generalised living in fear, traumatic events, and poor parental attachment, all of which were found to have adverse associations with chronic pain in adulthood.^29^^,^^64^^,^^59^^,^^61^^,^^62^^,^^65^^,^^56^^,^^58^^,^^78^^,^^66^^,^^69^^,^^89^^,^^88^^,^^84^^,^^62^, ^77^, ^81^, ^82^, ^91^ Studies focusing on adolescents with chronic pain have documented a dose response: the more adverse childhood experience a child experienced the more chronic pain and in a population of adolescents with pain the more adverse childhood experience the greater the mental health impact of anxiety and depression as well of fear of pain itself. These findings are consistent with those from this review of adult chronic pain.^98^^,^^99^

### Implications for research and practice

Despite the high prevalence of adverse childhood experiences documented in the included studies, not every person with chronic pain has experienced an adverse childhood experience, or *vice versa*. There was general agreement about the need for more prospective studies to determine the nature and extent of the links between adverse childhood experiences and chronic pain,^20^^,^^100^ and the reasons behind these. Data linkage, where adverse childhood experiences were previously recorded, or prospective cohort studies will provide more evidence.^101^^,^^102^ Most studies examined emotional, physical, and sexual abuse, but other traumatic events also need to be studied in detail. Emotional abuse was also noted to be both common and overlooked, requiring further research. This review demonstrates variability in acquisition of information on adverse childhood experiences. This review highlights the need to enquire about the possibility of adverse childhood experiences in those with chronic pain, and enquiring about current mental health symptoms. Understanding the potential for a history of adversity in people with chronic pain is an opportunity to consider management options.^31^^,^^36^^,^^48^ Further research into how this information is obtained should be undertaken to allow for consistency of approach. A history of childhood adversity should be considered in the assessment of chronic pain so that relevant management options can be determined (although further research into effective interventions is also required). Enquiring about childhood adversity has already been shown to be acceptable, although the sensitivity of this subject must always be respected and psychological safety maintained.^103^ Currently, National Institute for Health and Care Excellence in the UK do not recommend routinely assessing for adverse childhood experiences in chronic pain.^103^ Scottish Intercollegiate National Guidelines on management of chronic pain suggest a biopsychological assessment but the consistency of enquiring about adverse childhood experiences within clinical practice is unknown.^104^^,^^105^ An adverse childhood experience assessment has been recommended during a chronic pain assessment.^106^

## Conclusions

This review identified that a wide range of adverse childhood experiences are associated with the development, severity, and impact of chronic pain in adulthood, and that this association with adverse childhood experiences is consistent across a wide range of chronic pain conditions. The importance of adverse childhood experiences when assessing and managing chronic pain needs to be recognised and integrated into clinical practice. Further research on the underlying mechanisms of these associations is required, along with the assessment and management of childhood adversity in the context of chronic pain.

## Authors’ contributions

Planning and development of the review: KN, SEEM, LAC, BHS.

Initial plan for the review: KN.

Conception and design: KN, SEEM, DS.

Acquisition of data: KN, SEEM, DS.

Analysis and interpretation of data: KN, SEEM, DS.

Selection of tools for quality assessment: KN, SEEM.

Quality assessment: KN, SEEM, DS.

Identification of themes from included papers for narrative synthesis: KN, SEEM, DS.

Writing of the first draft of this paper: KN.

Overall research supervisors: LAC, BHS.

Development, thematic synthesis, interpretation, and overall conclusions: LAC, BHS.

Writing of the final version of the paper: KN, SEEM, DS, LAC, BHS.

Final approval of the version to be published: KN, SEEM, DS, LAC, BHS.

## Acknowledgements

We thank Mr Scott Macgregor, librarian, at The University of Dundee for his assistance.

## Declarations of interest

LAC was past editor of *British Journal of Anaesthesia* (2010–21), and is currently a member of the editorial board of the *BJA* and *BJA Open*. BHS was Scottish Government Lead Clinician for Chronic Pain (2014–2021) and is Speciality Adviser (Chronic Pain) to the Chief Medical Officer of Scotland. The authors have no other competing interests to declare.

## Funding

NHS Education for Scotland. KN was academic fellow from 2018 to 2022. SEEM was funded through an academic clinical fellowship from the Scottish Government's Chief Scientist Office (CSO grant number CAF_17_06). DS is a fellow on the Multimorbidity Doctoral Training Programme for Health Professionals, which is supported by the Wellcome Trust [grant number 223499/Z/21/Z].
