# Supplementary material for: What is the association between childhood adversity and subsequent chronic pain in adulthood? A systematic review
Source: BJA Open. 2023 Jun 7;6:100139. doi: 10.1016/j.bjao.2023.100139 (PMC10430872; doi:10.1016/j.bjao.2023.100139)
Supplement: Multimedia component 1 [file mmc1.docx]

| **Author** | **Country** | **Study type** | **Cohort size (controls)** | **Demography** | **Chronic Pain** | **Type of adversity studied** | **ACE Assessment Tool** |
| --- | --- | --- | --- | --- | --- | --- | --- |
| Achenbach J et al. 2022[1] | Germany | Case control | 151 (149) | Over 18 years. | Chronic Widespread Pain | Emotional/Physical/Sexual abuse | CTQ |
| Alhalal E et al. 2018 [2] | Saudi Arabia | Cross sectional | 299 | 100% F | Chronic pain | Emotional/Physical/Sexual abuse | Arabic CTQ [3] |
| Anda R et al. 2010 [4] | USA | Cross sectional | 17337 | 54% F 46% M mean age 56 | Self-reported 'Frequent Headache' | ACE | ACE questionnaire |
| Anno K et al. 2015[5] | Japan | Cross sectional | 760 | 62.4% F mean age 59.3 +/- 11.5 | Chronic pain across body | Parental attachment/affection/bonding | Parental Bonding Index |
| As-Sanie S et al. 2014 [6] | USA | Cross sectional | 273 | Mean age 34.8 | Chronic Pelvic Pain | Physical abuse | Physical and Sexual Abuse questionnaire |
| Bayram K et al. 2014 [7] | Turkey | Case control | 32 | F, mean age 39.1 +/- 9.1 | RA and FM | Emotional/Physical/Sexual abuse | CTQ |
| Bohn D et al. 2013 [8] | Germany | Cross sectional | 117 | 84% F | Fibromyalgia | Emotional/Physical/Sexual abuse | CTQ |
| Bottiroli S et al. 2018 [9] | Italy | Cross sectional | 331 | 81% F, mean age 40.1 +/- 11.0 | Chronic Migraine | Traumatic experience, Physical abuse and Emotional abuse | CTQ and Stressful life Events |
| Brown RC et al. 2018 [10] | Germany | Cross sectional | 2491 | 53.2% F mean age 48.3 | Musculoskeletal pain | Emotional/Physical/Sexual abuse | CTQ/Polo (Polytrauma) |
| Chiu CD et al. 2017 [11] | Hong Kong | Case control | 94 (47) | F, mean age 40.6+10.0 | Interstitial Cystitis/Bladder Pain | Sex/Emo/Phys/Violence (Witnessing and Domestic) | Brief Betrayal Trauma Survey |
| Coppens E et al. 2018 [12] | Belgium | Case control | 27(24) | F, FM 38.21(+/-10.45)  HC 38.59 (+/-10.29) | FM | Emotional/Physical/Sexual abuse | CTQ |
| Coppens E et al. 2017 [13] | Belgium | Cross sectional | 154 FM/CWP,83 Functional dyspepsia, 53 Achalasia) | F, mean age 42.46 | CWP and FM vs Functional Dyspepsia/achalasia | Emotional/Physical/Sexual abuse | CTQ |
| De Roa P et al. 2018 [14] | France | Cross sectional | 44 FM and 34 migraine | Mean age 45 +/- 12 | FM comparison to migraine | Emotional/Physical/Sexual abuse | CTQ and Holmes and Rahe Stressful events scale |
| Eitner S et al. 2009 [15] | Germany | Cross sectional | TMD- 50,  55- PDI/SOP (55) | Mixed M+F 80.6%  TMD mean age 46 PDI/SOP – 59.2  Control – mean age 54.5 | Oro-facial pain | Accident, illness, physical abuse | Physical well-being and traumatic experience scale |
| Eriksen AM et al. 2016 [16] | Norway | Cross sectional | 11130 | Adults | Chronic pain across body | Emotional/Physical/Sexual abuse | Independent Questionnaire |
| Fishbain DA et al. 2014 [17] | USA | Cross sectional | 2487 | Chronic Pain patients, acute pain patients, controls with no pain | Chronic pain across body | Emotional/Physical/Sexual abuse | Battery of Health Questionnaire |
| Fowler C et al. 2020 [18] | Ukraine | Cross sectional | 1720 | F 55% M 45% Mean age 46 | Pain | Adverse Childhood Experiences | ACE Questionnaire |
| Generaal E et al. 2016 [19] | Netherlands | Cross sectional | 1646 | Mixed M+F  Age 18-65 | Chronic pain | Emotional/Physical/Sexual abuse | Childhood Trauma Interview |
| Gerber MR et al. 2018 [20] | USA | Cross sectional | 22 | F age 30-76 | Fibromyalgia | Emotional/Physical/Sexual abuse | CTQ |
| Gonzalez A et al. 2012 [21] | Canada | Cohort | 1475 | Mixed, age 21-35 | Chronic back pain and migraine | Physical and sexual  abuse, parental mental health, poverty and family function | Childhood Experiences of Violence Questionnaire |
| Gunduz N et al. 2018 [22] | Turkey | Cross sectional | 52 FM, 35, MPS, (49) | F, mean age FM 42.65 MPS 41.97 HC 37.86 | Fibromyalgia/Myofascial pain | Emotional/Physical/Sexual abuse | CTQ |
| Harris HR et al. 2018 [23] | USA | Cross sectional | 60595 | F, age 25-42 | Endometriosis/Chronic pelvic pain | Physical and Sexual abuse | CTQ |
| Hart-Johnson T et al. 2012 [24] | USA | Cross sectional | 183 | Mixed adult cohort | Chronic Pain | Physical and Sexual abuse | Drossman Abuse Questionnaire |
| Hauser W et al. 2018 [25] | Germany | Cross sectional | 2425 | F 53.5% Mean age 50.8+/-17.5 | Chronic Non-Cancer pain | Emotional/Physical/Sexual abuse | Childhood Trauma Screener for ACE |
| Hauser W et al. 2015 [26] | Germany | Cross sectional | 71 | F 68% Mean age Germany 20.0 USA 51.9 | Fibromyalgia | Emotional/Physical/Sexual abuse | CTQ |
| Hauser W et al. 2012 [27] | Germany | Case control | 153(153) | 87.6% F mean age 50.3 | Fibromyalgia | Emotional/Physical/Sexual abuse | CTQ |
| Johnson B et al. 2020 | USA | Cross sectional | 181 | 80.1% Female | Chronic Pain | Sexual abuse, punishment and neglect | Childhood Abuse and trauma scale |
| Jones G et al. 2009 [28] | UK | Cohort | 7571 | Age 45 | Chronic pain | Hospitalisation, surgical operations, maternal separation, parental death, family difficulties | Survey |
| Kamiya Y et al. 2016 [29] | Ireland | Cross sectional | 8178 | Sex abuse cohort 56% female mean age 60.4 +/- 0.4. No abuse female 52% and mean age 64.2 +/- 0.1 | Widespread body pain, arthritis | Sexual abuse | 2 CSA questions and Stressful Life Events inventory |
| Karas et Al 2017 [30] | Turkey | Case control | 40(38) | Age not stated - female gender | Fibromyalgia | Emotional/Physical/Sexual abuse | CTQ |
| Kascakova N et al. 2020 [31] | Czech Republic | Cross sectional | 1800 | F 51.3% 48.7% M mean 46.61 (15-90) | Chronic Pain | Emotional/Physical/Sexual abuse | CTQ |
| Kascakova N et al. 2020 [32] | Czech Republic | Cross sectional | 1800 | F 51.3% 48.7% M mean 46.61 (15-90) | Migraine | Emotional/Physical/Sexual abuse | CTQ |
| Khandker M et al. 2014 [33] | USA | Case control | 215 (215) | F - age not stated. | Vulvodynia | Physical/Sexual abuse + Fear of danger | Independent Questionnaire |
| Liebermann C et al. 2018 [34] | Switzerland | Case control | 421 (421) | F - age not stated. | Endometriosis | Emotional/Physical/Sexual abuse | CTQ |
| Macedo B etc al. 2020 [35] | Brazil | Case control | 44 | Age 18-65 | Chronic Pain | Emotional/Physical/Sexual abuse | CTQ |
| Maatta J et al. 2019 [36] | Finland | Cross sectional | 43 | Age variable across cohorts. Mixed M+F | Chronic neuropathic pain | Emotional/Physical/Sexual abuse | Trauma and depression scale |
| McBeth J et al. 2015 [37] | UK | Cross sectional | 990 | 56% F - age 25 up | CF and CWP | Physical and Sexual abuse | Childhood Physical and Sex Abuse and Parental Bonding Questionnaire |
| McKernan LC et al. 2019 [38] | USA | Cross sectional | 202 | 79.7% F mean age 44.9 | IC/BPS | Emotional/Physical/Sexual abuse and family/household dysfunction. | Childhood Abuse and Trauma Scale |
| Muthuri SG et al. 2016 [39] | UK | Cross sectional | 1339 | F 52.1% Male 47.9% | Chronic widespread and regional pain | Serious illness before age 24 | Nil |
| Nacak Y et al. 2017 [40] | Germany | Case control | 65 | F 69.2% mean age 47.5 +/- 10.6 | Somatoform pain | Emotional/Physical/Sexual abuse and primary care giver attachment | CTQ |
| Naliboff BD et al. 2015 [41] | USA | Case control | 424 (417) | F 55% mean age 40.5 M 45% mean age 46.8 | Chronic Pelvic Pain | Traumatic events | Childhood Trauma Events Scale |
| Nault T et al 2016 [42] | USA | Cross sectional | 380 | F - mean age 50 | Chronic Pelvic Pain | Emotional/Physical/Sexual abuse and bullying. | Questions asked re bullying/abuse |
| Nicolson N et al. 2010 [43] | Holland | Cross sectional | 35(35) | FM mean age 53.5 OA mean age 58.4 | Fibromyalgia/Osteoarthritis | Emotional/Physical/Sexual abuse | CTQ |
| Ortiz R et al. 2016 [44] | USA | Cross sectional | 111 | Mean age 47.33 +/- 10.98 | Fibromyalgia | Emotional/Physical abuse | Physician interview |
| Piontek K et al 2021 | Germany | Cross sectional | 234 | F 55.98% Mean age 47.92. | Chronic Pelvic Pain syndrome | Childhood Adversity | ACE questionnaire |
| Poli-neto O et al. 2018 [45] | Brazil | Case control | 77(77) | 18 + | Chronic pelvic pain | Emotional/Physical/Sexual abuse | CTQ |
| Powers A et al. 2014 [46] | USA | Cross  Sectional | 814 | Mean age 41 | Long-term pain | Emotional/Physical/Sexual abuse | CTQ |
| Prangnell A et al. 2019 [47] | Canada | Cross sectional | 1459 | F 35% M 65% Av age 47.7 | Chronic Pain | Emotional/Physical/Sexual abuse | CTQ |
| Reuchlein B et al. 2016 [48] | Germany | Cross sectional | 1008 | Polish F 56.3% Mean age 38.7 +/-14.4 Germany F 50.0% Mean age 44.8 +/- 16.1 | Headache | Sexual/Emotional/Physical and family/household dysfunction and parental ill-health | Questions only |
| Riedl D et al. 2019 [49] | Austria | Cross sectional | 1480 | 52.1% F mean age 42.1 | Chronic Pain | Emotional/Physical/Sexual abuse | German Trauma Scale KERF |
| Saariaho TH et al. 2011 [50] | Finland | Cross sectional | 271 (918) | Mean age 47 Case - M 47% F 53% Control M 12% F 86% not known 2% | Chronic pain | Emotional abuse | Young Schema Question |
| Sachs-Ericsson N et al. 2017 [51] | USA | Cohort | 5001 | Mean age 43.03 | Painful Medical Conditions | ACEs | ACE questionnaire |
| Salonsalmi A et al. 2021 [52] | Finland | Cross sectional | 8140 | F 79.9%  Adult 40+ | Chronic Pain | Childhood Adversity | Questionnaire |
| Santo T et al. 2022 | Australia | Cross sectional | 1514 | 57 ( 13.72)  66% F 44% m | Chronic non cancer pain | Emotional Physical and sexual abuse, physical neglect, witnessing violence | Questionnaire |
| Schrepf A et al. 2018 [53] | USA | Case control | 421 (414) | F 53% mean age 43 +/- 15 | Chronic pelvic pain | Emotional/Physical/Sexual abuse and traumatic events | Childhood Trauma Events Scale |
| Scott KM et al. 2011 [54] | New Zealand | Cross sectional | 18303 | M+F cohort over 18 years old. | OA, Chronic spinal pain, Headache | Childhood adversity | Childhood adversity assessed |
| Smith BW et al. 2010 [55] | USA | Case control | 41(44) | F cohort Mean age 48.4 +/- 6.9 | Fibromyalgia | Traumatic events, road traffic accidents, natural disasters, witnessing death, child abuse) | Trauma experience questions asked |
| Stickley A et al. 2015 [56] | Japan | Cross sectional | 1740 | M+F cohort Mean age 51.2 | Chronic Pain | Emotional/Physical/Sexual abuse and family/household dysfunction and parental ill-health | Questions only |
| Taghian NR et al. 2020 | USA | Cross sectional | 372 | M 68.6%, mean age 37.7 +/- 14.2 | Chronic Pain | Physical and Sexual abuse | CTQ |
| Tesarz J et al. 2016 [57] | Germany | Cross sectional | 176(76) | 56.7(+/- 10) & 57.1(+/- 11.7) | Chronic back pain | Emotional/Physical/Sexual abuse | CTQ |
| Tietjen GE et al. 2010 part I [58] | USA | Cross sectional | 1348 | 88.1% F mean age 41 | Migraine | Emotional/Physical/Sexual abuse | CTQ |
| Tietjen GE et al. 2010 part II [59] | USA | Cross sectional | 1348 | 88.1% F mean age 41 | Migraine | Emotional/Physical/Sexual abuse | CTQ |
| Tietjen GE et al. 2010 part III [60] | USA | Cross sectional | 1348 | 88.1% F mean age 41 | Migraine | Emotional/Physical/Sexual abuse | CTQ |
| Varinen A et al. 2017 [61] | Finland | Cross sectional | 11924 | 62.1% F mean age - not stated | Fibromyalgia | Childhood adversity | Childhood Adversity Questionnaire |
| Varinen A et al 2019 [62] | Finland | Cross sectional | 11924 | 62.1% F mean age - not stated | Fibromyalgia | Bullying in childhood | Questionnaire on bullying |
| Waller E et al. [63] | Germany | Cross sectional | 34 | 100% F age not stated. | Fibromyalgia | Emotional/Physical/Sexual abuse and Parental attachment | Childhood Experience of Care and Abuse questionnaire. |
| Yeung EW et al. 2016 [64] | USA | Cross sectional | 179 | 89% F - mean age 51.83 | Fibromyalgia | Emotional/Physical/Sexual abuse | CTQ |
| You D et al. 2019 [65] | USA | Cross  sectional | 3073 | 72% F – mean age 18.8 | Chronic Pain Conditions | Emotional/Physical/Sexual abuse and Traumatic events | Questionnaire - independent |
| Ziadni M et al. 2020 [66] | USA | Cross  sectional | 742 | 66.3% F – mean age 49.7 | Chronic Pain | Perceived childhood neglect | Binary question |

*Female (F), male (M), fibromyalgia (FM), chronic fatigue (CF), osteoarthritis (OA), rheumatoid arthritis (RA), somatoform pain disorder (SPD), chronic pelvic pain (CPP), tempero-mandibular joint (TMJ), post-traumatic stress disorder (PTSD), interstitial cystitis/bladder pain syndrome (IC/BPS), childhood trauma questionnaire (CTQ), adverse childhood experiences (ACE).*

| **Table S2: Findings from studies included in narrative synthesis.** | | | | |
| --- | --- | --- | --- | --- |
| **Author** | **Chronic Pain Type** | **Study Type** | **Effect** | **Findings/Recommendations** |
| Tesarz J et al. 2016[57] | Chronic Back pain | Cross sectional studies | Childhood adversity was found to be more related to deep pain processing. Emotional abuse is related to pain sensitization. | Childhood adversity sensitizes the body to pain and affects somatosensory processes, emotional abuse is associated with enhanced pain. |
| Maatta et al.2009[36] | Chronic neuropathic pain | Cross sectional studies | Patients who had experienced much childhood adversity saw no benefit in their pain after spinal cord stimulation when compared with those with less adversity | Childhood adversity should be considered before and after spinal cord stimulation for chronic neuropathic pain and perhaps spinal cord stimulation might be best placed as part of a management plan alongside psychiatric intervention. |
| Gonzalez A et al. 2012 [21] | Chronic pain conditions | Cohort Study | Poor parental mental health and physical abuse in childhood were associated with many chronic diseases including chronic pain. Odds Ratio (OR) 1.66. | Early intervention is key prior to biological mechanisms affected by childhood adversity particularly in support for parental mental health, any maltreatment and family dysfunction. |
| Achenbach J et al. 2022[1] | CWP | Case-control | Hypocortisolism as a result of excess stress/burn out and allostatic load leads to pain and fatigue. Leptin was higher in those with more trauma and could cause stress induced hyper-analgesia. Dysregulation of the endocannaboid system may lead to hormonal resistance. | Chronic stress and childhood adversity lead to a disordered endocrine profile. Further studies on endocrine hormone profiles, including 24 hr measurements. |
| Jones G et al. 2009 [28] | Chronic widespread pain (CWP) | Cohort Study | Those who experience physical trauma and social adversity are at 50-100% greater risk of CWP, particularly maternal separation (Relative risk (RR) 2.0), time in care (RR1.7) or road traffic accidents. (RR 1.6) | In order to avoid detrimental long-term complications future research should focus on biological mechanisms. |
| Alhalal E et al. 2018 [67] | Chronic Pain | Cross sectional studies | Severity of childhood abuse had no direct impact on chronic pain, but it did on PTSD which in turn mediates effects between chronic pain and childhood abuse. | Abuse severity, childhood and interpersonal violence impact mental health which in turn can cause chronic pain - childhood adversity and mental health assessments should be done and treated accordingly. |
| Anno K et al. 2015 [5] | Chronic Pain | Cross sectional studies | Those who experience affectionless parenting are more likely to have chronic pain. Emotional neglect and lower levels of parental care and over-protection was linked to chronic pain. | Poor parental bonding has a detrimental effect on health potential leading to chronic pain. |
| Brown R et al. 2018 [10] | Chronic Pain | Cross sectional studies | More childhood abuse led to greater levels of pain above anxiety and depression. Emotional abuse had a large effect on all pains, physical neglect and bullying related to pain. | Levels of pain in women were affected by all adversity however pain was only affected in men by physical/sexual abuse. |
| Fishbain D et al. 2014[17] | Chronic pain | Cross sectional studies | Sexual abuse was not found to be related to pain. | Those with and without pain can have experienced childhood sexual abuse. |
| Fowler C et al. 2020 [18] | Chronic pain | Cross sectional studies | The highest prevalence of condition was chronic pain even 1 or 2 adversities correlated with more pain. | More research needed on mediating factors, children should be safe in their families, in a safe environment. |
| Eriksen AM et al. 2016[16] | Chronic Pain | Cross sectional studies | Those who experienced any childhood violence had more chronic pain sites and more severe pain. | Patients with chronic pain of unknown origin should be asked about any childhood adversity |
| Generaal E et al. 2016 [19] | Chronic Pain | Cross sectional studies | Childhood adversity was related to the presence and severity of chronic pain. No relationship found between trauma and brain-derived neurotrophic factor found | Early life adversity and ongoing life stress were found to be related chronic pain diagnosis and impact |
| Hart-Johnson T et al. 2012 [24] | Chronic Pain | Cross sectional studies | 67% reported physical or sexual abuse or being threatened. Childhood abuse before 14 had greater impact on pain, disability and quality of life. Severe sexual abuse was related to worse pain related disability. | Previous adversity can make dealing with existing pain harder to cope with. Pain treatment may be less effective if trauma history not assessed. Health practitioners might need specific training. |
| Johnson B et al. 2020[68] | Chronic Pain | Cross sectional | All childhood trauma correlated borderline personality disorder (BPD) and chronic pain. ACE is a risk factor for BPD, trauma is a predictor of pain. Adult trauma does not show the same consistent relationship for more chronic pain. | Patients with Chronic pain should be assessed for BPD. Referring patients with BPD and chronic pain for help with emotional regulation could be beneficial. Ask about childhood adversity in those who have chronic pain so therapy can be directed. |
| Kamiya Y et al. 2016 [29] | Chronic Pain | Cross sectional studies | Chronic pain related to childhood sexual abuse however, once anxiety and depression controlled for, this was not found to be significant. This was after all other childhood adversity controlled for. | Childhood sexual abuse is association with anxiety, depression, worry, loneliness as well as chronic pain. |
| Kascakova N et al.2020[31] | Chronic Pain | Cross sectional studies | Those with anxiety and chronic pain had higher prevalence of emotional and physical abuse. Maternal neglect of child impacting neurobiology. | Prevention in the form of education for populations on negative impact of this adversity. More research on emotional abuse is needed. |
| Macedo B et al 2020 [35] | Chronic Pain | Cross sectional studies | Those with adversity in childhood had more severe depression with depression and pain, they were more likely to have experienced emotional neglect. | Emotional neglect as a child puts you at higher risk of experiencing chronic pain as an adult, up to 4 times. Larger study needed. |
| Prangnell et al 2019[47] | Chronic pain | Cross sectional studies | Childhood emotional abuse means a greater susceptibility to chronic pain in adulthood in vulnerable population of people who take drugs. | Management of Chronic pain should look for early life adversities and earlier interventions considered - trauma informed chronic pain practice needs to be available to vulnerable groups. |
| Riedl D et al. 2019 [49] | Chronic pain | Cross sectional studies | More than 4 types of childhood adversity were indicative of physical disease chronic pain was the most prevalent. Domestic violence later in life is related to childhood violence. | Early intervention to prevent childhood adversity is crucial, we should be better at asking about domestic violence in primary care. |
| Saariaho TH et al. 2011[50] | Chronic pain | Cross sectional studies | Those with chronic pain may have on going negative thoughts from early emotional abuse, impacting their ability to function whilst experiencing chronic pain. Emotional abuse can be harder to detect. | Negative processing of thought life can lead to greater disability and severity in managing chronic pain. Specific psychological therapy  should be considered. |
| Salonsalmi A et al.  2021 [52] | Chronic pain | Cross sectional studies | Childhood adversity is linked to chronic pain. Bullying, financial problems as a child and childhood illness is linked to chronic pain which is more disabling. | Investing in the well-being of children and families may well be key to reduced chronic pain in later life. |
| Stickley A et al. 2015 [56] | Chronic pain | Cross sectional studies | Childhood adversity in the form of abuse or family dysfunction is related to chronic pain. | Training those who work with children to identify adversity might help to reduce chronic pain in adult life |
| Taghian NR et al. 2020 [69] | Chronic Pains | Cross sectional studies | In those with substance mis-use physical and sexual abuse as a child led to twice the likelihood of experiencing chronic pain than those without substance misuse and this increased with age. Pain severity and catastrophization were also higher for those who have suffered physical abuse, those with sexual abuse had higher catastrophization and pain interference. | Patients with Substance use disorder should be asked about this type of trauma so that interventions to mitigate impact can be put in place to help. |
| You D et al. 2019[65] | Chronic pain | Cross sectional studies | The greater the adversities experienced the higher risk for chronic pain in young adults | Perhaps the number of childhood adverse events is more important than evaluating the type of adversity whilst investing childhood trauma in those with chronic pain. |
| Ziadni MS et al. 2020 [66] | Chronic pain | Cross sectional studies | Child adversity and perceived injustice (feelings of blame/loss) correlates with poor physical functionality and negative affect for adults impacted. | Further studies are needed but emotional distress and management aiming at emotional well-being should be considered. |
| Santo T et al 2022[70] | Chronic Non-Cancer Pain (CNCP) | Cross sectional studies | Childhood trauma causes long term harm.  In those with CNCP, a history of childhood trauma meant an increased risk of opioid misuse disorder as well as mental health disorders and substance misuse. | Assessment of those with chronic pain and history of childhood adversity may reduce development of opioid use disorder. Trauma informed services should be available. Further research is required. |
| Sachs-Ericsson N et al. 2017 [51] | Chronic pain conditions | Cohort Study | Verbal, sexual abuse, parental loss early in life and poor parental mental health found to be associated with painful medical conditions - i.e., chronic pains. | The greater the extent of childhood adversity the higher risk for mental health disorders and chance of developing medical conditions that are painful, the ACE impact on pain was higher for those reporting less mood/anxiety issues regardless of mental health the more 'ACEs' the more pain related conditions experienced |
| Schrepf A et al. 2018 [53] | Chronic pelvic  pain | Case Control Studies | Those with abuse +/- bullying predicted greater levels of pelvic pain. | Patients with pelvic pain should be screened for childhood adversities, bullying, anxiety and depression. |
| Naliboff BD et al. 2015[41] | Chronic pelvic pain | Case Control Studies | More early childhood adversity and life-long adversity in those with chronic pelvic pain. | Women, compared to men with pelvic pain noted more childhood adversity and had more detrimental symptoms of discomfort and pain. |
| Poli-neto O et al. 2018 [45] | Chronic pelvic pain | Case Control Studies | Those with CPP experience more types of abuse than health controls. | Chronic pelvic pain is complex, but childhood adversity may be involved in the pathophysiology. Mental health and social stress may also contribute. |
| As-Sanie S et al. 2014 [6] | Chronic Pelvic Pain | Cross sectional studies | No pain severity difference noted between those who experience childhood abuse and not, adolescent and adult abuse was linked. More pain related disability with abuse as adolescent/adult. | History of adversity is a greater indicator of mental health disorder rather than pain; patients should be assessed for life-time abuse and depression if they have chronic pelvic pain. |
| Nault T et al 2016 [42] | Chronic Pelvic Pain | Cross sectional studies | Women with history of sexual and physical abuse with or without bullying experience more pelvic pain. | Bullying and a history of adversity should be considered in those with chronic pelvic pain to enable specific therapeutic plans to be in place, assessment for anxiety and depression should also be done. |
| Piontek K et al 2021[71] | Chronic Pelvic Pain | Cross sectional | Emotional maltreatment was most common in those with CPPS. ACE linked to more depression and higher pain intensity. Pain perception may be impacted by depression in those with ACE. | Enquiring about childhood adversity in those with Chronic pelvic pain is advised. Identification and treatment of depression is also key. |
| Scott KM et al. 2011 [54] | Chronic spinal pain and Headache | Cross sectional studies | Several adversities such as physical abuse, parental loss, violence sexual abuse and general neglect were associated with spinal pain and headache, the more adversities experienced the greater the association with pain. | Childhood adversity and early diagnosed mental health conditions are independently related to a variety of chronic conditions in adult life including spinal pain and headaches. Prospective studies are needed. |
| Mcbeth J et al. 2015 [37] | CWP + Chronic Fatigue | Cross sectional studies | Those with CWP+CF and anxiety + depression were more likely to have experienced childhood adversity. Early parental loss, physical abuse and psychological abuse were twice as common in the CWP group. | CWP does not necessarily mean someone has experience childhood neglect however this should be explored, and anxiety/depression considered in a routine manner. |
| Muthuri SG et al. 2016 [39] | CWP + CF | Cross sectional studies | Those who experience serious illness before the age of 25 were at higher risk of enduring chronic widespread pain at age 68. | Is CWP part of pain/distress and somatic syndrome, more support is needed for those who experience serious illness early in life. |
| Liebermann C et al. 2018 [34] | Endometriosis | Case Control Studies | Patients with endometriosis experienced more emotional abuse and neglect, sexual abuse and inconsistency in childhood. Other situational adversity i.e., parent in prison not significant. | Childhood adversity should be enquired about in those with endometriosis. |
| Harris HR et al. 2018 [23] | Endometriosis | Cohort Study | Sexual and physical abuse separately linked to a higher risk of endometriosis. 21% of those with endometriosis has experience both adversities. | Further research required - Is pain in endometriosis linked to childhood adversity? |
| Coppens E et al. 2018 [12] | Fibromyalgia (FM) | Case Control Studies | Those with history of adversity had higher levels of cortisol, reduced cortisol response to stress and increase subjectivity of stress were found in those with FM independent of history of trauma | The reactivity to the stressor in those with fibromyalgia was less. |
| Hauser W et al. 2012 [27] | Fibromyalgia | Case Control Studies | FM patients report more severe childhood adversity than those with OA (but not a great difference) and in turn they had more depressive symptoms | The possibility of childhood adversity should be assessed in those who have fibromyalgia and those with other chronic pain conditions |
| Smith B et al. 2010 [55] | Fibromyalgia | Case Control Studies | Those with FM were 3x more likely to have experience abuse as a child. Those with FM also perceived more stress which was related to worse physical and mental health. Adult abuse mediates the affects. | FM is a 'stress disorder' related to increase exposure to stress and higher perceived stress levels. Interventions to reduced impact of stress and cycle of abuse are needed. |
| Bohn D et al.2013 [8] | Fibromyalgia | Cross sectional studies | Nearly 50% showed possible dissociation disorder, 95.7% probable somatoform disorder and 73.5% depression. High rates of childhood maltreatment in FM. Emotional abuse was related to somatoform severity | Adversity in childhood might be pathological in the development of FM symptoms - therapy allowing these events to be processed should be analysed. |
| De Roa P et al.2008 [14] | Fibromyalgia | Cross sectional studies | The most common adversity was emotional neglect. Family stress can lead to parents being unable to care properly for their children. Those with FM reported more lack of affection from parents than migraine cohort. | Could a constant state of hypervigilance due to emotional neglect lead to fibromyalgia? The impact of life experience and stress should play a part in FM management. It should be a syndrome and stress/coping mechanisms and behaviours assessed. |
| Gerber MR et al. 2018[20] | Fibromyalgia | Cross sectional studies | Sexual abuse and emotional neglect which in turn led to higher sexual trauma as an adult and PTSD. | Women with FM might benefit from trauma focussed care and biopsychosocial approach - more work is needed to determine effective interventions. |
| Gunduz N et al. 2018 [22] | Fibromyalgia | Cross sectional studies | FM group had more than double the percentage of most types of abuse and neglect than the control group (physical neglect as exception) | Significant relationship between childhood trauma and mental health disorders - both should be analysed |
| Hauser W et al. 2015[26] | Fibromyalgia | Cross sectional studies | Adversity is not required to develop FM though is risk factor for this, mental health mediates adversity and FM symptoms. | More research is needed. Screening for mental health in FM patients is advised. |
| Karas H et al. 2017[30] | Fibromyalgia | Cross sectional studies | Dissociative symptoms and childhood trauma are common in those with fibromyalgia stated to cause a detrimental impact to worsen pain experience | Psychiatry specialists could be involved in specific management for patients with fibromyalgia and a history of childhood trauma. |
| Varinen A et al. 2017 [61] | Fibromyalgia | Cross sectional studies | Family conflict, financial difficulties, being fearful of family member, alcohol and divorce as childhood adversity were all related to fibromyalgia. | Prevention of childhood adversity is crucial. |
| Varinen A et al 2019 [62] | Fibromyalgia | Cross sectional studies | Bullying in childhood is related to fibromyalgia, the direct cause is unknown. | Bullying should be considered as a factor in the development of fibromyalgia as a childhood adversity, prevention of bullying is key. |
| Waller E et al.2016 [63] | Fibromyalgia | Cross sectional studies | 50% were reported to have unresolved experience of loss and abuse. 38.8% experienced emotional neglect and 23.4% parental antipathy. | Those with FM might have not yet processed parental attachment issues and experiences of adversity. How this impacts management is not yet known. Disclosure of such history is not thought to impact outcomes. |
| Yeung EW et al. 2016 [64] | Fibromyalgia | Cross sectional studies | Those with childhood adversity and fibromyalgia, had flattened cortisol patterns which was connected to more emotional symptoms and daily pain. | More research on the type and kind of adversity on pain will enable better management options for those who suffer with FM. |
| Bayram K et al. 2014 [7] | Fibromyalgia | Case Control Studies | More emotional abuse and higher anxiety + depression in FM vs rheumatoid arthritis (RA) but RA had higher rates than health controls also. Sexual abuse history led to highest anxiety and depression scores. | Patients with RA and FM should be screened for childhood adversity and symptoms of anxiety/depression. |
| Coppens E et al 2017 [13] | FM/CWP | Cross sectional studies | Those with pain were 4 times more likely to report a childhood adversity than those without. No difference in pain severity in those with pain and adversity experienced. | PTSD connected adversity with severity of pain experienced. Those with pain were 6 times more likely to meet criteria for PTSD. Patients should be screened for PTSD. |
| Ortiz R et al. 2016 [44] | FM | Cross sectional studies | Low levels of adversity found in FM but those with adversity and FM were more sensitive to pain and reduced tolerance for pain. | Those with adversity experience FM differently. Childhood adversity changes biology in those with FM. |
| Nicolson N et al. 2010 [72] | FM/OA | Cross sectional studies | Different types of adversity had different impacts on cortisol levels. Pain and adversity did not show correlation with cortisol level hypothesised variations. | Could Chronic Pain cause cortisol irregularities independently? Those with FM should be asked about adversity. Does the heterogeneity of FM cause the variability in results? |
| Anda R et al. 2010 [4] | Headaches | Cross sectional studies | The greater number of childhood adversity (higher ACE score) the greater number of headaches experienced. | Greater understanding of the link between headaches and childhood adversity might lead to different management options for frequent headaches in the future. |
| Bottiroli S et al. 2018 [9] | Headaches | Cross sectional studies | Emotional and physical trauma was prevalent in those with all types of headache. | Emotional trauma should be investigated, as often overlooked. Any form of trauma may impact these conditions. |
| Reuchlein B et al. 2016 [48] | Headaches | Cross sectional studies | Physical and Emotional neglect together were associated with headache development, other adversities were not linked, a father with chronic pain was predictive of headache in women. | Childhood adversity emotional and physical neglect should be considered in those with headache, but other childhood adversities do not appear to be related. |
| McKernan LC et al. 2019 [38] | IC/Bladder pain | Cross sectional studies | Those with pelvic pain issues were more likely to have PTSD when had been exposed to childhood trauma. | PTSD should be considered in this population and management should be trauma informed, ensuring that investigations/procedures do no cause further trauma. |
| Chiu CD et al. 2017 [11] | Interstitial Cystitis Bladder Pain | Case Control Studies | Higher rates of all neglects than control group, statistical significance for physical abuse, reported more trauma by those close to them. | Childhood adversity might not be pathogenic but might contribute to its continuation. |
| Powers A et al. 2014 [46] | Long-term Pain | Cross sectional studies | PTSD is associated with great amount of pain and lower level of bodily function. Emotional dysregulation needs to play a part. | More awareness of the connection between childhood adversity/PTSD and long-term pain needs to be known. Emotional regulation management may improve pain in PTSD/long-term pain. |
| Kascakova N et al. 2020 [32] | Migraine | Cross sectional studies | Exposure to childhood adversity/poor attachment led to higher likelihood of reporting migraine. | Those with chronic migraine should be screened for childhood adversity and attachment insecurity. |
| Tietjen GE et al. 2010 part I [58] | Migraine | Cross sectional studies | Emotional abuse and neglect reported by many in cohort, many experienced all types of abuse. | Childhood adversity was common in those with migraine, maltreatment was associated with depression and anxiety the more adversity the greater chance of mental health disorder. |
| Tietjen GE et al. 2010 part II [59] | Migraine | Cross sectional studies | Emotional abuse was associated with daily headache and migraine, it was associated with headache severity and earlier age of migraine development. | Emotional abuse is less recognised but might be involved in the pathophysiology of migraine/headache. |
| Tietjen GE et al. 2010 [60] | Migraine and Chronic pain | Cross sectional studies | Migraine and emotional abuse and physical neglect had higher number of pain conditions than those with this type of maltreatment. | In those with migraine, childhood adversity might lead to further chronic pain conditions. |
| Hauser W et al. 2018 [25] | Non cancer CP | Cross sectional studies | No statistical significance found between chronic non cancer pain and childhood adversity. | Prospective studies assessing children who have lived through trauma should be done to determine if specific pain syndromes are related. |
| Eitner S et al. 2009 [15] | Oro-facial pain | Cross sectional studies | Oro-facial pain comparison – those with TMJ pain had highest incidence of accidents or illness as a child. Childhood trauma is associated with chronic dental pain. | More research on the connection between chronic dental pain and childhood adversity is required. |
| Nacak Y et al. 2017 [40] | Somatoform Pain disorder | Case Control Studies | Those with SPD experienced more sexual, emotional and physical abuse. 60% of those with SPD had insecure attachment to primary care giver. | Further studies to be able to identify patients with this condition is needed, more information on the aetiology would help to guide therapeutic options. |
| Khandker M et al. 2014 [33] | Vulvodynia | Case Control Studies | Patients with pain experienced had double the amount of severe abuse than controls, and far greater percentage lived in fear at school, neighbourhood and at home. | Chronic stress could be important in the psychobiological aspects of pathogenesis of vulvodynia, (along with coping with a mood disorder) |

| **Table S3: Quality appraisal of cross-sectional studies using AXIS tool.** | | | | | | | | | | | | | | | | | | | | |
| --- | --- | --- | --- | --- | --- | --- | --- | --- | --- | --- | --- | --- | --- | --- | --- | --- | --- | --- | --- | --- |
| **Author** | **Q1** | **Q2** | **Q3** | **Q4** | **Q5** | **Q6** | **Q7** | **Q8** | **Q9** | **Q10** | **Q11** | **Q12** | **Q13** | **Q14** | **Q15** | **Q16** | **Q17** | **Q18** | **Q19** | **Q20** |
| Alhalal E et al. 2018 [67] | Y | Y | Y | Y | Y | Y | Y | Y | Y | Y | Y | Y | N | Y | Y | Y | Y | Y | N | Y |
| Anda R et al. 2010 [4] | Y | Y | Y | Y | Y | Y | Y | Y | Y | Y | Y | Y | Y | Y | Y | Y | Y | Y | Y | N |
| Anno K et al. 2015 [5] | Y | Y | Y | Y | Y | Y | Y | Y | Y | Y | Y | Y | DK | Y | DK | Y | Y | Y | N | Y |
| As-Sanie S et al. 2014 [6] | Y | Y | Y | Y | Y | Y | Y | Y | Y | Y | Y | Y | N | NA | DK | Y | Y | Y | N | Y |
| Bohn D et al.2013 [8] | Y | Y | Y | Y | Y | Y | Y | Y | Y | Y | Y | Y | N | NA | Y | Y | Y | Y | N | Y |
| Bottiroli S et al. 2018[9] | Y | Y | Y | Y | Y | Y | N | Y | Y | Y | Y | Y | N | N | DK | Y | Y | Y | N | Y |
| Brown R et al. 2018 [10] | Y | Y | Y | Y | Y | Y | Y | Y | Y | Y | Y | Y | N | NA | DK | Y | Y | Y | N | Y |
| Coppens E et al. 2017 [13] | Y | Y | Y | Y | Y | Y | N | Y | Y | Y | Y | Y | N | NS | DK | Y | Y | Y | N | DK |
| De Roa P et al. 2008[14] | Y | Y | Y | Y | Y | Y | N | Y | Y | Y | Y | Y | N | NA | DK | Y | Y | Y | N | N |
| Eitner S et al. 2009 [15] | Y | Y | Y | Y | Y | Y | N | Y | N | Y | N | N | NA | NA | DK | DK | Y | N | N | Y |
| Fishbain D et al. 2014[17] | Y | Y | Y | Y | Y | Y | Y | Y | Y | Y | Y | Y | DK | NA | DK | Y | Y | Y | DK | DK |
| Fowler C et al. 2020[18] | Y | Y | Y | Y | Y | Y | N | Y | Y | Y | Y | Y | N | NA | Y | Y | Y | Y | N | DK |
| Eriksen AM et al. 2016 [16] | Y | Y | Y | Y | Y | Y | Y | Y | DK | Y | Y | Y | N | NA | Y | Y | Y | Y | N | Y |
| Generaal E et al. 2016 [73] | Y | Y | Y | Y | Y | Y | Y | Y | Y | Y | Y | Y | Y | Y | DK | Y | Y | Y | N | Y |
| Gerber MR et al. 2018[20] | Y | Y | Y | Y | Y | Y | N | Y | Y | N | N | Y | N | NA | NA | Y | Y | Y | N | Y |
| Gunduz N et al. 2018 [22] | Y | Y | Y | Y | Y | Y | DK | Y |  | Y | Y | Y | DK | NA | Y | Y | Y | Y | DK | Y |
| Hauser W et al. 2016[74] | Y | Y | N | Y | Y | Y | N | Y | Y | Y | Y | Y | N | NA | DK | DK | Y | Y | DK | Y |
| Hauser W et al. 2018[25] | Y | Y | N | Y | Y | Y | N | Y | Y | Y | Y | Y | DK | DK | NA | DK | Y | Y | N | Y |
| Hart-Johnson T et al. 2012[24] | Y | Y | Y | Y | Y | Y | Y | Y | Y | Y | Y | Y | N | NA | DK | DK | Y | Y | N | N |
| Johnson et al. 2020 [68] | Y | Y | N | Y | Y | Y | N | Y | Y | Y | Y | Y | DK | NA | Y | Y | Y | Y | Y | Y |
| Kamiya Y et al. 2016 [29] | Y | Y | Y | Y | Y | Y | DK | Y | DK | Y | N | Y | N | NA | DK | Y | Y | Y | N | Y |
| Karas H et al. 2017 [75] | Y | Y | Y | Y | N | Y | N | Y | Y | Y | Y | N | N | NA | DK | Y | Y | Y | N |  |
| Kascakova N et al. 2020 [76] | Y | Y | Y | Y | Y | Y | Y | Y | Y | Y | Y | Y | N | NA | Y | Y | Y | Y | N | Y |
| Kascakova N et al. 2020 [77] | Y | Y | N | Y | Y | Y | Y | Y | Y | Y | Y | Y | N | Y | Y | Y | Y | Y | N | Y |
| Maatta et al. 2009 [36] | Y | Y | Y | Y | Y | Y | N | Y | Y | Y | Y | Y | N | NA | Y | Y | Y | Y | N | Y |
| Macedo B et al. 2020 [78] | Y | Y | N | Y | Y | Y | N | Y | Y | Y | Y | Y | Y | N | Y | Y | Y | Y | N | Y |
| Mcbeth J et al. 2015 [37] | Y | Y | Y | Y | Y | Y | Y | Y | Y | Y | Y | Y | Y | Y | Y | Y | Y | Y | N | Y |
| McKernan LC et al. 2019 [38] | Y | Y | Y | Y | Y | y | DK | Y | Y | Y | Y | Y | N | NA | DK | Y | Y | Y | N | Y |
| Muthuri SG et al. 2016 [39] | Y | Y | Y | Y | Y | Y | Y | Y | Y | Y | N | NA | Y | Y | Y | Y | Y | Y | N | Y |
| Nault T et al. 2016[42] | Y | Y | Y | Y | Y | Y | N | Y | N | Y | Y | Y | DK | NA | Y | Y | Y | Y | N | Y |
| Nicolson N et al. 2010[72] | Y | Y | Y | Y | Y | Y | N | Y | Y | Y | Y | Y | N | NA | Y | Y | Y | Y | N | Y |
| Ortiz R et al. 2016 [44] | Y | Y | Y | Y | Y | Y | N | Y | Y | Y | Y | Y | N | NA | DK | Y | Y | Y | N | Y |
| Piontek et al. 2020 [71] | Y | Y | N | Y | Y | Y | N | Y | Y | Y | Y | Y | DK | N | Y | Y | Y | Y | Y | Y |
| Powers A et al. 2014[46] | Y | Y | Y | Y | Y | Y | N/A | Y | Y | Y | Y | Y | N/A | N/A | Y | Y | Y | Y | N | Y |
| Prangnell et al. 2019 [79] | Y | Y | Y | Y | Y | Y | N | Y | Y | Y | Y | DK | N | NA | DK | Y | Y | Y | N | Y |
| Reuchlein B et al. 2016 [48] | Y | Y | Y | Y | Y | Y | N | Y | Y | Y | Y | Y | NA | NA | Y | Y | Y | Y | N | Y |
| Riedl D et al. 2019 [49] | Y | Y | Y | Y | Y | Y | Y | Y | Y | Y | Y | Y | N | NA | Y | Y | Y | Y | N | Y |
| Saariaho TH et al. 2011 [50] | Y | Y | Y | Y | Y | Y | N | Y | Y | Y | Y | Y | N | NA | DK | Y | Y | Y | N | Y |
| Santo et al. 2021[70]a | Y | Y | N | Y | Y | Y | NA | Y | Y | Y | Y | Y | NA | NA | Y | Y | Y | Y | N | Y |
| Scott KM et al. 2011 [54] | Y | Y | Y | Y | Y | Y | N | Y | Y | Y | Y | Y | NA | NA | DK | Y | Y | Y | N | Y |
| Stickley A et al. 2015 [56] | Y | Y | Y | Y | Y | Y | N | Y | Y | Y | Y | Y | N | N | DK | Y | Y | Y | N | Y |
| Taghian et al. 2020 [69] | Y | Y | N | Y | Y | Y | Y | Y | Y | Y | Y | Y | Y | Y | Y | Y | Y | Y | N | Y |
| Tesarz J et al. 2016 [80] | Y | Y | Y | Y | Y | Y | NA | Y | Y | Y | Y | Y | N | NA | DK | Y | Y | Y | N | Y |
| Tietjen GE et al. 2010 part I [58] | Y | Y | N | Y | Y | Y | N | Y | Y | Y | Y | Y | N | NA | Y | Y | Y | Y | N | Y |
| Tietjen GE et al. 2010 part II [59] | Y | Y | N | Y | Y | Y | N | Y | Y | Y | Y | Y | N | NA | Y | Y | Y | Y | N | Y |
| Tietjen GE et al. 2010 part III [60] | Y | Y | N | Y | Y | N | N | Y | Y | Y | Y | Y | DK | NA | DK | Y | Y | Y | DK | Y |
| Varinen A et al. 2017[61] | Y | Y | Y | Y | Y | Y | Y | Y | Y | Y | Y | Y | N | NA | Y | Y | Y | Y | N | Y |
| Varinen A et al 2019[62] | Y | Y | Y | Y | Y | Y | Y | Y | Y | Y | Y | Y | N | NA | Y | Y | Y | Y | N | Y |
| Waller E et al.2016 [63] |  | Y | Y | Y | Y | Y | Y | Y | Y | Y | Y | Y | N | NA | Y | Y | Y | Y | N | Y |
| Yeung EW et al. 2016 [81] | Y | Y | Y | Y | Y | Y | N | Y | Y | Y | Y | Y | N | NA | DK | Y | Y | Y | N | Y |
| You D et al. 2020 [82] | Y | Y | N | Y | Y | Y | N | Y | Y | Y | Y | Y | N | NA | Y | Y | Y | Y | N | Y |
| Ziadni MS et al. 2020 [83] | Y | Y | Y | Y | Y | Y | N | Y | Y | Y | Y | Y | N | NA | Y | Y | Y | Y | N | Y |

*Q1 Were the aims/objectives of the study clear? Q2 Was the study design appropriate for the stated aim(s)? Q3 Was the sample size justified? Q4 Was the target/reference population clearly defined? Q5 Was the sample frame taken from an appropriate population base so that it closely represented the target/reference population under investigation? Q6 Was the selection process likely to select subjects/participants that were representative of the target/reference population under investigation? Q7 Were measures undertaken to address and categorise non-responders? Q8 Were the risk factor and outcome variables measured appropriate to the aims of the study? Q9 Were the risk factor and outcome variables measured correctly using instruments/measurements that had been trialled, piloted or published previously? Q10 Is it clear what was used to determined statistical significance and/or precision estimates? Q11 Were the methods (including statistical methods) sufficiently described to enable them to be repeated? Q12 Were the basic data adequately described? Q13 Does the response rate raise concerns about non-response bias? Q14 If appropriate, was information about non-responders described? Q15 Were the results internally consistent? Q16 Were the results presented for all the analyses described in the methods? Q17 Were the authors' discussions and conclusions justified by the results? Q18 Were the limitations of the study discussed? Q19 Were there any funding sources or conflicts of interest that may affect the authors’ interpretation of the results? Q20 Was ethical approval or consent of participants attained?*

| **Table S4: Quality appraisal of cohort studies using SIGN tool.** | | | | |
| --- | --- | --- | --- | --- |
|  | **Gonzalez A et al. 2012 [21]** | **Harris HR et al. 2018 [23]** | **Jones G et al. 2009 [84]** | **Sachs-Ericsson N et al. 2017 [51]** |
| Appropriate question | Y | y | Y | Y |
| Groups comparable | Y | DNA | DNA | Y |
| Number of people | Y | Y | Y | Y |
| Prior outcome assessed | DNA | Y | DNA | Y |
| Percentage recruited | 41.50% | 48% | 71.50% | 38% |
| Comparison | DNA | Y | Can’t say | Y |
| Outcome defined | Y | Y | Y | Y |
| Outcome blind to exposure | DNA | DNA | DNA | DNA |
| Exposure status noted | Can’t say | N | Y | Can’t say |
| Reliable method | DNA | Y | Y | Y |
| Exposure level assessed | DNA | Y | Y | DNA |
| Main confounders noted | Can’t say | Y | Y | Y |
| Confidence Intervals | Y | Y | Y | Y |
| Bias noted | Y | Y | Y | Y |
| Bias minimized | High Quality | High Quality | High Quality | High Quality |
| Clear evidence | Y | Y | Y | Y |
| Results applicable | Y | Y | Y | Y |

| **Table S5: Quality appraisal of case-control studies using SIGN tool.** | | | | | | | | | | | | |
| --- | --- | --- | --- | --- | --- | --- | --- | --- | --- | --- | --- | --- |
|  | **Achenbach J et al. 2022 [1]** | **Bayram K et al. 2014 [7]** | **Chiu CD et al. 2017 [11]** | **Coppens E et al. 2018 [85]** | **Hauser W et al. 2012[27]** | **Khandker M et al. 2014 [33]** | **Liebermann C et al. 2018 [86]** | **Nacak Y et al. 2017 [40]** | **Naliboff BD et al. 2015 [41]** | **Poli-Neto et al. 2018 [87]** | **Schrepf A et al. 2018 [53]** | **Smith BW et al. 2010 [55]** |
| Focussed question | Y | Y | Y | Y | Y | Y | Y | Y | Y | Y | Y | Y |
| Comparable populations | Can’t say | Can't say | Y | Y | Y | Y | Y | Y | Y | Y | Y | Y |
| Same exclusion criteria | Y | Y | Y | Y | Y | Y | Y | Y | Can't say | Y | Y | Y |
| Percentage of case and control | 50.3% + 49.7% | 67%+33% | 67.4% + 32.6% | 52.8%+47.2% | 50%+50% | 42.5% + 57.5% | 50%+50% | 50%+50% | 50.4%+49.6% | 50%+50% | 50.2%+ 49.6% | 48.2%+51.8% |
| Comparison made | Y | Y | Y | Y | Y | Y | Y | Y | Y | Y | Y | Y |
| Case clearly defined | Y | Y | Y | Y | Y | Y | Y | Y | Y | Y | Y | Y |
| Cases are not controls | N | Y | Y | N | Y | Y | Y | Y | Y | Y | Y | Y |
| Primary exposure not known | DNA | DNA | Y | DNA | Can't say | Y | DNA | Can't say | DNA | Y | Can't say | Can't say |
| Exposure noted | Y | Y | Y | Y | Y | Y | Y | Y | N | Y | Y | Y |
| Confounders documented | Y | Y | Y | Can't say | Y | Y | Y | Y | N | Y | Y | Y |
| Bias noted | N | Y | Y | Can't say | Y | Y | Y | Y | Y | N | Can't say | Can't say |
| Confidence interval | Y | N | N | Y | N | Y | N | Y | Y | Y | Y | N |
| Bias minimized | Low quality | Acceptable | High quality | High quality | High quality | Acceptable | High quality | High quality | Acceptable | Acceptable | Acceptable | Acceptable |
| Clear evidence noted | N | Y | N | Y | Y | Y | Y | Y | Can't say | Can't say | Y | Y |
| Guidelines applicable | N | Y | N | Y | Y | Y | Y | Y | Y | Y | Y | Y |

**References**

1. Achenbach, J., et al., *Neurohumoral Profiles and Childhood Adversity of Patients with Multisomatoform Disorder and Pain as the Leading Bodily Symptom.* Disease markers, 2022. **2022**.

2. Alhalal, E., et al., *Factors mediating the impacts of child abuse and intimate partner violence on chronic pain: A cross-sectional study 11 Medical and Health Sciences 1117 Public Health and Health Services 17 Psychology and Cognitive Sciences 1701 Psychology.* BMC Women's Health, 2018. **18**(1).

3. Al-Zahrani, A.H., *Child abuse & neglect: its forms, causes and consequences in the Kingdom of Saudi Arabia*. 2004, University of Edinburgh.

4. Anda, R., et al., *Adverse childhood experiences and frequent headaches in adults.* Headache, 2010. **50**(9): p. 1473-81.

5. Anno, K., et al., *Paternal and maternal bonding styles in childhood are associated with the prevalence of chronic pain in a general adult population: the Hisayama Study.* BMC Psychiatry, 2015. **15**: p. 181.

6. As-Sanie, S., et al., *History of abuse and its relationship to pain experience and depression in women with chronic pelvic pain.* Am J Obstet Gynecol, 2014. **210**(4): p. 317.e1-317.e8.

7. Bayram, K. and A. Erol, *Childhood Traumatic Experiences, Anxiety, and Depression Levels in Fibromyalgia and Rheumatoid Arthritis.* Noro Psikiyatr Ars, 2014. **51**(4): p. 344-349.

8. Bohn, D., et al., *The association among childhood maltreatment, somatic symptom intensity, depression, and somatoform dissociative symptoms in patients with fibromyalgia syndrome: a single-center cohort study.* Journal of Trauma & Dissociation, 2013. **14**(3): p. 342-358.

9. Bottiroli, S., et al., *Traumatic Experiences, Stressful Events, and Alexithymia in Chronic Migraine With Medication Overuse.* Frontiers in Psychology, 2018. **9**.

10. Brown, R.C., et al., *Associations of adverse childhood experiences and bullying on physical pain in the general population of Germany.* J Pain Res, 2018. **11**: p. 3099-3108.

11. Chiu, C.D., et al., *Childhood trauma perpetrated by close others, psychiatric dysfunction, and urological symptoms in patients with interstitial cystitis/bladder pain syndrome.* Journal of Psychosomatic Research, 2017. **93**: p. 90-95.

12. Coppens, E., et al., *Cortisol and Subjective Stress Responses to Acute Psychosocial Stress in Fibromyalgia Patients and Control Participants.* Psychosom Med, 2018. **80**(3): p. 317-326.

13. Coppens, E., et al., *Prevalence and impact of childhood adversities and post-traumatic stress disorder in women with fibromyalgia and chronic widespread pain.* Eur J Pain, 2017. **21**(9): p. 1582-1590.

14. De Roa, P., et al., *Subjective Experiences and Sensitivities in Women with Fibromyalgia: A Quantitative and Comparative Study.* Pain Res Manag, 2018. **2018**: p. 8269564.

15. Eitner, S., et al., *Biopsychosocial correlations in patients with chronic oro-facial pain. Part II. Experiences of pain and dramatic events before the 16th year of life.* J Oral Rehabil, 2009. **36**(6): p. 408-14.

16. Eriksen, A.M., et al., *Childhood violence and adult chronic pain among indigenous Sami and non-Sami populations in Norway: a SAMINOR 2 questionnaire study.* Int J Circumpolar Health, 2016. **75**: p. 32798.

17. Fishbain, D.A., et al., *Exploration of affirmation of childhood molestation (sexual abuse) in chronic pain patients, acute pain patients, community patients with pain and community nonpatients without pain.* Pain Pract, 2014. **14**(6): p. 515-25.

18. Fowler, C., et al., *Adult correlates of adverse childhood experiences in Ukraine.* Child Abuse & Neglect, 2020. **107**: p. 104617.

19. Generaal, E., et al., *The brain-derived neurotrophic factor pathway, life stress, and chronic multi-site musculoskeletal pain.* Molecular Pain, 2016. **12**.

20. Gerber, M.R., et al., *Experience of Childhood Abuse and Military Sexual Trauma Among Women Veterans with Fibromyalgia.* J Gen Intern Med, 2018. **33**(12): p. 2030-2031.

21. Gonzalez, A., et al., *Childhood and family influences on depression, chronic physical conditions, and their comorbidity: findings from the Ontario Child Health Study.* J Psychiatr Res, 2012. **46**(11): p. 1475-82.

22. Gunduz, N., et al., *Psychiatric comorbidity and childhood trauma in fibromyalgia syndrome.* Turkish Journal of Physical Medicine and Rehabilitation, 2018. **64**(2): p. 91-99.

23. Harris, H.R., et al., *Early life abuse and risk of endometriosis.* Hum Reprod, 2018. **33**(9): p. 1657-1668.

24. Hart-Johnson, T. and C.R. Green, *The impact of sexual or physical abuse history on pain-related outcomes among blacks and whites with chronic pain: gender influence.* Pain Med, 2012. **13**(2): p. 229-42.

25. Hauser, W., et al., *The association of adverse childhood experiences and of resilience with chronic noncancer pain in the German adult population - A cross-sectional survey.* Eur J Pain, 2018.

26. Häuser, W., et al., *Self-reported childhood maltreatment, lifelong traumatic events and mental disorders in fibromyalgia syndrome: a comparison of US and German outpatients.* Clinical and experimental rheumatology, 2015. **33**(1 0 88): p. S86.

27. Häuser, W., et al., *Is the association of self-reported childhood maltreatments and adult fibromyalgia syndrome attributable to depression? A case control study.* Clin Exp Rheumatol, 2012. **30**(6 Suppl 74): p. 59-64.

28. Jones, G.T., C. Power, and G.J. Macfarlane, *Adverse events in childhood and chronic widespread pain in adult life: Results from the 1958 British Birth Cohort Study.* Pain, 2009. **143**(1-2): p. 92-6.

29. Kamiya, Y., V. Timonen, and R.A. Kenny, *The impact of childhood sexual abuse on the mental and physical health, and healthcare utilization of older adults.* Int Psychogeriatr, 2016. **28**(3): p. 415-22.

30. Karas, H., et al., *The relationship of childhood trauma, dissociative experiences and depression with pain in female patients with fibromyalgia: A cross- sectional study.* Dusunen Adam, 2017. **30**(2): p. 86-94.

31. Kascakova, N., et al., *The Unholy Trinity: Childhood Trauma, Adulthood Anxiety, and Long-Term Pain.* International Journal of Environmental Research and Public Health, 2020. **17**(2).

32. Kascakova, N., et al., *When a Head Is about to Burst: Attachment Mediates the Relationship Between Childhood Trauma and Migraine.* International Journal of Environmental Research and Public Health, 2020. **17**(12).

33. Khandker, M., et al., *Is chronic stress during childhood associated with adult-onset vulvodynia?* J Womens Health (Larchmt), 2014. **23**(8): p. 649-56.

34. Liebermann, C., et al., *Maltreatment during childhood: a risk factor for the development of endometriosis?* Hum Reprod, 2018.

35. Macedo, B.B.D., et al., *Child Abuse and Neglect as Risk Factors for Comorbidity Between Depression and Chronic Pain in Adulthood.* J Nerv Ment Dis, 2019. **207**(7): p. 538-545.

36. Maatta, J., et al., *High Level of Childhood Trauma Predicts a Poor Response to Spinal Cord Stimulation in Chronic Neuropathic Pain.* Pain Physician, 2019. **22**(1): p. E37-E44.

37. McBeth, J., et al., *Common and unique associated factors for medically unexplained chronic widespread pain and chronic fatigue.* J Psychosom Res, 2015. **79**(6): p. 484-91.

38. McKernan, L.C., et al., *Posttraumatic stress disorder in interstitial cystitis/bladder pain syndrome: Relationship to patient phenotype and clinical practice implications.* Neurourol Urodyn, 2019. **38**(1): p. 353-362.

39. Muthuri, S.G., et al., *Chronic physical illness in early life and risk of chronic widespread and regional pain at age 68: evidence from the 1946 British birth cohort.* Pain, 2016. **157**(10): p. 2382-9.

40. Nacak, Y., et al., *Insecure attachment style and cumulative traumatic life events in patients with somatoform pain disorder: A cross-sectional study.* J Psychosom Res, 2017. **103**: p. 77-82.

41. Naliboff, B.D., et al., *Widespread Psychosocial Difficulties in Men and Women With Urologic Chronic Pelvic Pain Syndromes: Case-control Findings From the Multidisciplinary Approach to the Study of Chronic Pelvic Pain Research Network.* Urology, 2015. **85**(6): p. 1319-27.

42. Nault, T., et al., *Does a history of bullying and abuse predict lower urinary tract symptoms, chronic pain, and sexual dysfunction?* International Urology and Nephrology, 2016. **48**(11): p. 1783-1788.

43. Nicolson, N.A., et al., *Childhood maltreatment and diurnal cortisol patterns in women with chronic pain.* Psychosom Med, 2010. **72**(5): p. 471-80.

44. Ortiz, R., et al., *Quantifying the influence of child abuse history on the cardinal symptoms of fibromyalgia.* Clin Exp Rheumatol, 2016. **34**(2 Suppl 96): p. S59-66.

45. Poli-Neto, O.B., et al., *History of childhood maltreatment and symptoms of anxiety and depression in women with chronic pelvic pain.* J Psychosom Obstet Gynaecol, 2018. **39**(2): p. 83-89.

46. Powers, A., et al., *Childhood abuse and the experience of pain in adulthood: the mediating effects of PTSD and emotion dysregulation on pain levels and pain-related functional impairment.* Psychosomatics, 2014. **55**(5): p. 491-499.

47. Prangnell, A., et al., *The Impact of Childhood Emotional Abuse on Pain Interference Among People with Chronic Pain who Inject Drugs in Vancouver, Canada.* Pain Med, 2020. **21**(4): p. 704-713.

48. Reuchlein, B., et al., *Childhood Adversities and Adult Headache in Poland and Germany.* PLoS One, 2016. **11**(2): p. e0148162.

49. Riedl, D., et al., *Violence from childhood to adulthood: The influence of child victimization and domestic violence on physical health in later life.* J Psychosom Res, 2019. **116**: p. 68-74.

50. Saariaho, T.H., et al., *Early maladaptive schemas in Finnish adult chronic pain patients and a control sample.* Scand J Psychol, 2011. **52**(2): p. 146-53.

51. Sachs-Ericsson, N.J., et al., *When Emotional Pain Becomes Physical: Adverse Childhood Experiences, Pain, and the Role of Mood and Anxiety Disorders.* J Clin Psychol, 2017. **73**(10): p. 1403-1428.

52. Salonsalmi, A., et al., *Contributions of childhood adversities to chronic pain among mid-life employees.* Scand J Public Health, 2021: p. 1403494820981509.

53. Schrepf, A., et al., *Adverse Childhood Experiences and Symptoms of Urologic Chronic Pelvic Pain Syndrome: A Multidisciplinary Approach to the Study of Chronic Pelvic Pain Research Network Study.* Annals of Behavioral Medicine, 2018. **52**(10): p. 865-877.

54. Scott, K.M., et al., *Association of childhood adversities and early-onset mental disorders with adult-onset chronic physical conditions.* Arch Gen Psychiatry, 2011. **68**(8): p. 838-44.

55. Smith, B.W., et al., *Traumatic Events, Perceived Stress and Health in Women with Fibromyalgia and Healthy Controls.* Stress and Health, 2010. **26**(1): p. 83-93.

56. Stickley, A., A. Koyanagi, and N. Kawakami, *Childhood adversities and adult-onset chronic pain: Results from the World Mental Health Survey, Japan.* Eur J Pain, 2015. **19**(10): p. 1418-27.

57. Tesarz, J., et al., *Altered pressure pain thresholds and increased wind-up in adult patients with chronic back pain with a history of childhood maltreatment: a quantitative sensory testing study.* Pain, 2016. **157**(8): p. 1799-1809.

58. Tietjen, G.E., et al., *Childhood maltreatment and migraine (part I). Prevalence and adult revictimization: a multicenter headache clinic survey.* Headache, 2010. **50**(1): p. 20-31.

59. Tietjen, G.E., et al., *Childhood maltreatment and migraine (part II). Emotional abuse as a risk factor for headache chronification.* Headache, 2010. **50**(1): p. 32-41.

60. Tietjen, G.E., et al., *Childhood maltreatment and migraine (part III). Association with comorbid pain conditions.* Headache, 2010. **50**(1): p. 42-51.

61. Varinen, A., et al., *The relationship between childhood adversities and fibromyalgia in the general population.* J Psychosom Res, 2017. **99**: p. 137-142.

62. Varinen, A., et al., *The association between bullying victimization in childhood and fibromyalgia. Data from the nationwide Finnish health and social support (HeSSup) study based on a sample of 64,797 individuals.* J Psychosom Res, 2019. **117**: p. 48-53.

63. Waller, E., et al., *Unresolved trauma in fibromyalgia: A cross-sectional study.* Journal of Health Psychology, 2016. **21**(11): p. 2457-2465.

64. Yeung, E.W., M.C. Davis, and M.C. Ciaramitaro, Ann Behav Med, 2016. **50**(1): p. 87-97.

65. You, D.S., et al., *Cumulative Childhood Adversity as a Risk Factor for Common Chronic Pain Conditions in Young Adults.* Pain Med, 2019. **20**(3): p. 486-494.

66. Ziadni, M.S., et al., *Perceived Injustice Mediates the Relationship Between Perceived Childhood Neglect and Current Function in Patients with Chronic Pain: A Preliminary Pilot Study.* J Clin Psychol Med Settings, 2020.

67. Alhalal, E., et al., *Factors mediating the impacts of child abuse and intimate partner violence on chronic pain: a cross-sectional study.* BMC Womens Health, 2018. **18**(1): p. 160.

68. Johnson, B.N., et al., *Exploring the links among borderline personality disorder symptoms, trauma, and pain in patients with chronic pain disorders.* Journal of psychosomatic research, 2020. **135**: p. 110164.

69. Taghian, N.R., et al., *Associations between childhood abuse and chronic pain in adults with substance use disorders.* Substance Use & Misuse, 2021. **56**(1): p. 87-92.

70. Santo Jr, T., et al., *Exposure to childhood trauma increases risk of opioid use disorder among people prescribed opioids for chronic non-cancer pain.* Drug and alcohol dependence, 2022. **230**: p. 109199.

71. Piontek, K., et al., *Depression partially mediates the association of adverse childhood experiences with pain intensity in patients with chronic pelvic pain syndrome: results from a cross-sectional patient survey.* Pain medicine, 2021. **22**(5): p. 1174-1184.

72. Nicolson, N.A., et al., *Childhood maltreatment and diurnal cortisol patterns in women with chronic pain.* Psychosomatic medicine, 2010. **72**(5): p. 471-480.

73. Generaal, E., et al., *The brain-derived neurotrophic factor pathway, life stress, and chronic multi-site musculoskeletal pain.* Molecular pain, 2016. **12**: p. 1744806916646783.

74. Häuser, W., *Fibromyalgia syndrome: Basic knowledge, diagnosis and treatment.* Medizinische Monatsschrift fur Pharmazeuten, 2016. **39**(12): p. 504-511.

75. Karas, H., et al., *The relationship of childhood trauma, dissociative experiences and depression with pain in female patients with fibromyalgia: a cross-sectional study.* Dusunen Adam The Journal of Psychiatry and Neurological Sciences, 2017. **30**(2): p. 86.

76. Kascakova, N., et al., *When a head is about to burst: Attachment mediates the relationship between childhood trauma and migraine.* International Journal of Environmental Research and Public Health, 2020. **17**(12): p. 4579.

77. Kascakova, N., et al., *The unholy trinity: childhood trauma, adulthood anxiety, and long-term pain.* International journal of environmental research and public health, 2020. **17**(2): p. 414.

78. Macedo, B.B.D., et al., *Child abuse and neglect as risk factors for comorbidity between depression and chronic pain in adulthood.* The Journal of nervous and mental disease, 2019. **207**(7): p. 538-545.

79. Prangnell, A., et al., *The impact of childhood emotional abuse on pain interference among people with chronic pain who inject drugs in Vancouver, Canada.* Pain medicine, 2020. **21**(4): p. 704-713.

80. Tesarz, J., et al., *Altered pressure pain thresholds and increased wind-up in adult patients with chronic back pain with a history of childhood maltreatment: a quantitative sensory testing study.* Pain, 2016. **157**(8): p. 1799-1809.

81. Yeung, E.W., M.C. Davis, and M.C. Ciaramitaro, *Cortisol Profile Mediates the Relation Between Childhood Neglect and Pain and Emotional Symptoms among Patients with Fibromyalgia.* Ann Behav Med, 2016. **50**(1): p. 87-97.

82. You, D.S., et al., *Cumulative childhood adversity as a risk factor for common chronic pain conditions in young adults.* Pain Medicine, 2019. **20**(3): p. 486-494.

83. Ziadni, M.S., et al., *Perceived injustice mediates the relationship between perceived childhood neglect and current function in patients with chronic pain: A preliminary pilot study.* Journal of clinical psychology in medical settings, 2021. **28**(2): p. 349-360.

84. Jones, G.T., C. Power, and G.J. Macfarlane, *Adverse events in childhood and chronic widespread pain in adult life: Results from the 1958 British Birth Cohort Study.* Pain, 2009. **143**(1-2): p. 92-96.

85. Coppens, E., et al., *Cortisol and subjective stress responses to acute psychosocial stress in fibromyalgia patients and control participants.* Psychosomatic Medicine, 2018. **80**(3): p. 317-326.

86. Liebermann, C., et al., *Maltreatment during childhood: a risk factor for the development of endometriosis?* Human Reproduction, 2018. **33**(8): p. 1449-1458.

87. Poli-Neto, O.B., et al., *History of childhood maltreatment and symptoms of anxiety and depression in women with chronic pelvic pain.* Journal of Psychosomatic Obstetrics & Gynecology, 2018. **39**(2): p. 83-89.
